# Dual-Phase PET-CT to Differentiate [^18^F]Fluoromethylcholine Uptake in Reactive and Malignant Lymph Nodes in Patients with Prostate Cancer

**DOI:** 10.1371/journal.pone.0048430

**Published:** 2012-10-31

**Authors:** Daniela E. Oprea-Lager, Andrew D. Vincent, Reindert J. A. van Moorselaar, Winald R. Gerritsen, Alfons J. M. van den Eertwegh, Jonas Eriksson, Ronald Boellaard, Otto S. Hoekstra

**Affiliations:** 1 Department of Nuclear Medicine and PET Research, VU University Medical Center, Amsterdam, The Netherlands; 2 Department of Biometrics, Antoni van Leeuwenhoek Hospital, The Netherlands Cancer Institute, Amsterdam, The Netherlands; 3 Department of Urology, VU University Medical Center, Amsterdam, The Netherlands; 4 Department of Oncology, St Radboud University Medical Center, Nijmegen, The Netherlands; 5 Department of Oncology, VU University Medical Center, Amsterdam, The Netherlands; 6 Department of Medicinal Chemistry, Organic Pharmaceutical Chemistry, Uppsala University, Uppsala, Sweden; Stanford, United States of America

## Abstract

**Purpose:**

To investigate whether time-trends of enhanced [^18^F]Fluoromethylcholine ([^18^F]FCH) in lymph nodes (LN) of prostate cancer (PCa) patients can help to discriminate reactive from malignant ones, and whether single time point standardized uptake value (SUV) measurements also suffice.

**Procedures:**

25 PCa patients with inguinal (presumed benign) and enlarged pelvic LN (presumed malignant) showing enhanced [^18^F]FCH uptake at dual-phase PET-CT were analyzed. Associations between LN status (benign versus malignant) and SUV_max_ and SUV_meanA50_, determined at 2 min (early) and 30 min (late) post injection, were assessed. We considered two time-trends of [^18^F]FCH uptake: type A (SUV early > SUV late) and type B (SUV late ≥ SUV early). Histopathology and/or follow-up were used to confirm the assumption that LN with type A pattern are benign, and LN with type B pattern malignant.

**Results:**

Analysis of 54 nodes showed that LN status, time-trends, and ‘late’ (30 min p.i.) SUV_max_ and SUV_meanA50_ parameters were strongly associated (*P*<0.0001). SUV_max_ relative difference was the best LN status predictor. All but one inguinal LN showed a decreasing [^18^F]FCH uptake over time (pattern A), while 95% of the pelvic nodes presented a stable or increasing uptake (pattern B) type.

**Conclusions:**

Time-trends of enhanced [^18^F]FCH uptake can help to characterize lymph nodes in prostate cancer patients. Single time-point SUV measurements, 30 min p.i., may be a reasonable alternative for predicting benign versus malignant status of lymph nodes, but this remains to be validated in non-enlarged pelvic lymph nodes.

## Introduction

Choline-based PET has proven value in several neoplasms, and especially in prostate cancer [1], [2]. [^18^F]FCH PET-CT seems to become a useful imaging tool to solve the clinical problem of rising serum prostate-specific antigen (PSA) after initially treated prostate cancer [3]–[5], with PET-CT sensitivity being proportionally related to the PSA level [6], [7].

Choline is the precursor of phosphatidylcholine (PC), an essential phospholipid of cell membranes. The phosphorylation process is catalyzed by choline kinase (CK) [2]. In prostate cancer, enhanced choline uptake is explained by both increased mitogenic activity as well as up regulation of CK [8]. However, the signal is not tumor specific [9], [10]. Since biopsy of presumed regional lymph node metastases is often not trivial due to their localization in prostate cancer, false positive readings may induce serious problems in the clinical context.

Both [^18^F] and [^11^C]-labeled choline derivates have been developed and studied as possible metabolic imaging tools in the detection of primary PCa, regional LN and distant metastases [11], [12]. The main advantages of [^18^F] over [^11^C]-labeled tracers are the longer half-life (110 min versus 20 min) and a better spatial resolution due to the shorter positron range of ^18^F. However, [^18^F]choline is excreted in urine [13] and this can compromise the interpretation of the pelvic area. Since [^18^F]FCH is rapidly cleared from the blood pool, acquisition protocols have been designed with imaging of the pelvis prior to bladder filling (within minutes after injection), followed by a whole body scan after e.g. 30 min [1], [14]–[16] – the dual-phase protocol. Patterns of tracer uptake as a function of time have shown to be helpful in discriminating intraprostatic tracer uptake [8], [15] as well as sites of suspected haematogeneous metastases [17].

It was suggested that increasing or stable [^18^F]FCH uptake over time (3–7 min versus 30–60 min after injection) was compatible with malignancy, while a decreasing tracer uptake is associated with benign status [8], [15], [17]. For lymph node assessment this hypothesis has not been validated. Beheshti et al. [8] described an interesting early [^18^F]FCH wash-out pattern of false-positive LN in the preoperative setting of 130 patients with high and intermediate risk for disseminated prostate cancer. This phenomenon was deemed highly important for the differentiation of malignant versus benign nodes. However, the study could not support this finding with a statistically relevant number of lesions.

**Table 1:** Patient characteristics.

Total patients (N = 25)	Pelvic group (N = 17)	Combined pelvic &inguinal (N = 1)	Inguinal group (N = 7)
Mean age (years)	63 (range: 50–80)	63	63 (range: 57–68)
Mean serum PSA (ng/ml) at diagnosis	51	27	18
Mean serum PSA (ng/ml) at PET time	38	37	17
Gleason score (number of patients)			
<7	4	–	2
7	7	–	4
>7	6	1	1
Previous therapy: number of patients (indication)			
EBRT (±ADT ±PLND)	5 (3×I_1_+2×I_3_)	–	2 (2×I_1_)
EBRT + RP	2 (2×I_1_)	–	1 (I_1_)
RP	1 (I_1_)	–	2 (2×I_1_)
ADT	1 (I_1_)	–	1 (I_3_)
BT	1 (I_1_)	–	–
NA	7 (7×I_2_)	1 (I_3_)	1 (I_2_)

*N* number, *EBRT* external -beam radiation therapy, *ADT* anti-androgen therapy, *PLND* pelvic lymph node dissection, *RP* radical prostatectomy, *BT* brachytherapy, *NA* not applicable, *I_1_*: PSA relapse after therapy, *I_2_*: newly diagnosed prostate cancer, *I_3_*: staging patients with suspected oligometastases from prostate cancer.

In the present study we investigated whether time-trends of enhanced [^18^F]FCH uptake in lymph nodes can help to discriminate between benign and malignant sites, and we explored whether single time point SUV measurements may also suffice.

**Table 2:** SUV metrics of lymph nodes as a function of time.

Standard Uptake Value	Benign	Malignant	Total
(SUV)	N = 17	N = 37	N = 54
*Maximum*			
Early (2 min p.i.)			
Median	2	3.5	3
(Range)	(1.4–8.4)	(1.4–10)	(1.4–10)
Late (30 min p.i.)			
Median	1.4	4.3	3.2
(Range)	(0.95–4.8)	(2.2–11)	(0.95–11)
Difference			
Median	−0.64	0.44	0
(Range)	(−3.6– −0.08)	(−1.4–3.8)	(−3.6–3.8)
Relative Difference			
Median	−0.32	0.084	0
(Range)	(−0.54– −0.055)	(−0.25–1.4)	(−0.54–1.4)
*MeanA50*			
Early (2 min p.i.)			
Median	1.5	2.6	2.3
(Range)	(1.1–5.6)	(1.2–7.2)	(1.1–7.2)
Late (30 min p.i.)			
Median	1.1	3.4	2.6
(Range)	(0.75–3.4)	(1.8–8.1)	(0.75–8.1)
Difference			
Median	−0.42	0.31	0.03
(Range)	(−2.2–0.02)	(−1–2.7)	(−2.2–2.7)
Relative Difference			
Median	−0.29	0.11	0.013
(Range)	(−0.49–0.019)	(−0.22–1.4)	(−0.49–1.4)

*Difference* = SUV_late_ – SUV_early._

*Relative difference* = (SUV_late_ – SUV_early_)/SUV_early_.

## Materials and Methods

### Patients

Formal ethical approval for performing this retrospective study was obtained from the Medical Ethics Committee of the VU University Medical Center, Amsterdam, The Netherlands (approval date June 2012, reference 2012/254). This approval states that written informed consent from participants is wavered since the study does not fall within the scope of the Medical Research Involving Human Subjects Act (section 16.2 WMO, 26^th^ February 1998).

**Figure 1:**
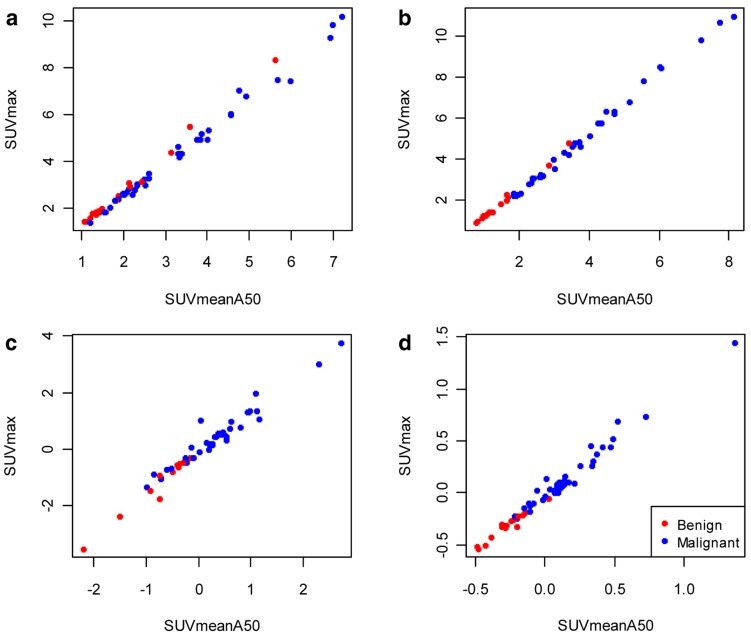
Comparison of SUV_max_ and SUV_meanA50_. **a** Early = 2 min p.i.; **b** Late = 30 min p.i.; **c** Difference = SUV_late_ – SUV_early_; **d** Relative difference = (SUV_late_ – SUV_early_)/SUV_early_. At the early scan time point, SUV_max_ = 1.40*SUV_mean_ –0.07(Benign) –0.27(Malignant); versus a reduction of −0.07 in the intercept at the late (30 min p.i.) assessment.

**Table 3:** Optimal thresholds for different SUV measures.

Standard Uptake Value (SUV)	Threshold	Patterntype	Ben	Mal	Sensitivity(95% CI)	Specificity(95% CI)	AUC(95% QR)
SUV_max_							
Late (30 min p.i.)	2.32	A	15	3	92	88	0.93
		B	2	34	(78–98)	(64–99)	(0.88–0.94)
Difference	−0.31	A	16	6	84	94	0.90
		B	1	31	(68–94)	(71–100)	(0.87–0.97)
Relative Difference	−0.19	A	16	2	95	94	0.98
		B	1	35	(82–99)	(71–100)	(0.95–0.99)
SUV_meanA50_							
Late (30 min p.i.)	1.66	A	15	0	100	88	0.95
		B	2	37	(91–100)	(64–99)	(0.91–0.95)
Difference	−0.18	A	16	7	81	94	0.89
		B	1	30	(65–92)	(71–100)	(0.84–0.98)
Relative Difference	−0.15	A	16	3	92	94	0.97
		B	1	34	(78–98)	(71–100)	(0.92–0.99)

*Pattern type:* A = SUV_early_ > SUV_late_; B = SUV_early_ ≤ SUV_late._

*Ben* benign, *Mal* malignant, *CI* confidence interval, *AUC* area under the ROC curve, *QR* quartile range.

*Difference* = SUV_late_ – SUV_early._

*Relative difference* = (SUV_late_ – SUV_early_)/SUV_early._

We retrospectively studied [^18^F]FCH PET-CT scans of 66 consecutive patients with prostate cancer (median age, 63 years; range, 50–80 years), performed at the VU University Medical Center, Amsterdam, The Netherlands, between January 2009 and March 2011. Three main clinical indications (I) for PET-CT were: I_1_. PSA relapse in previously treated PCa (n = 39); I_2_. newly diagnosed PCa (n = 16) and I_3_. staging of patients with suspected oligometastases (typically skeletal, identified with other imaging modalities), in newly diagnosed or already treated PCa (n = 11).

Patient characteristics [age, Gleason score (Gl), serum PSA at diagnosis and at the time of performing the PET-CT] were gathered, including the date and the type of previous therapy [e.g. radical prostatectomy (RP), external-beam radiation therapy (EBRT), brachytherapy (BT), pelvic lymph node dissection (PLND), anti-androgen therapy (ADT) or the combination of these].

Inclusion criteria were: dual-phase [^18^F]FCH PET-CT performed in patients with histopathologically proven PCa; enhanced [^18^F]FCH uptake in any inguinal nodes and in pelvic LN with a short axis diameter ≥8 mm, visible at early and/or late PET scan. Patients with multiple malignancies (e.g. one or more other types of carcinoma apart from the PCa) were excluded.

PSA relapse, suspected to be associated with residual or recurrent disease, was defined as a serum concentration level above 0.2 ng/ml, after RP and after the combination of RP with other types of therapy. A rising PSA level >2 ng/ml above the nadir value in patients treated by means of EBRT was considered suspect for persistent or recurrent disease [18]. Increased serum PSA was confirmed by two consecutive exams for all patients. The maximal time interval between performing the PET-CT scan and the last PSA determination was 14 days.

Based on histology, the primary PCa was classified as low (Gl <7), intermediate (Gl = 7) and high grade (Gl>7), according to the modified Gleason Grading System [19].

### Lymph Node Classification

We classified lymph nodes as benign or malignant using the following approach: inguinal lymph nodes with enhanced [^18^F]FCH uptake were considered benign since the prostatic lymph node drainage pattern does not include inguinal nodes [20]–[22]. Pelvic nodes with a short axis diameter ≥8mm were classified as malignant [23]. Additional confirmation was obtained using histopathology (whenever feasible), and with clinical follow-up of 6–12 months in all patients. Follow-up consisted of PSA measurements over time (as above mentioned) and/or evaluation of other imaging (i.e., contrast-enhanced abdominopelvic CT, pelvic MRI). We applied the following radiological criteria to classify change [24]: an increase by 30% versus initial size as progression, a decrease by 30% as regression, and intermediate values or no change in size as stable.

Therefore, malignancy was defined as a positive histopathological result; radiologically confirmed progression in size of the pertaining lymph nodes; decrease or normalization of serum PSA and radiological response after therapy, providing these were the only abnormal findings on the initial PET-CT scan; decrease or normalization of serum PSA with nodal regression after RT, with the RT field including the site of the suspected LN.

### Synthesis of [^18^F]FCH

[^18^F]FCH was synthesized according to the methods proposed by DeGrado et al. [1], [25] and Iwata et al. [26], with minor modifications and by use of automated modules built in-house [27]. In short, cyclotron produced [^18^F]fluoride was reacted with dibromomethane, the formed [^18^F]fluorobromomethane was purified and used in the alkylation of 2-(dimethylamino)ethanol to obtain 2.6±0.9 GBq [^18^F]FCH after semi-preparative high performance liquid chromatography (HPLC) purification, reformulation in aqueous 0.9% NaCl and sterile filtration.

The radiochemical purity was >99.5% and no chemical impurities were detected as assessed by analytical radio/ultra-violet-HPLC (UV-HPLC). Residual concentrations of dibromomethane (0–8 ppm) and 2-(dimethylamino)ethanol (95±40 ppm) were determined by flame ionization detector - gas chromatography (FID-GC). Absence of bacterial endotoxins in the product was confirmed by an Endosafe portable test system (PTS) reader (Charles River) and all samples were tested for sterility.

### PET-CT Imaging

All studies were performed on a Gemini TOF-64 PET-CT scanner (Philips Medical Systems, Best, The Netherlands) with an axial field per view of 18 cm. Low-dose CT (LD-CT) was collected using a beam current of 30 to 50 mAs at 120 keV. CT was reconstructed using an image matrix size of 512×512 resulting in voxel sizes of 1.17×1.17 mm and a slice thickness of 5 mm. For PET, data were reconstructed by means of a raw action ordered subset expectation maximization algorithm using default reconstruction parameters. Time of flight (TOF) information was used during reconstruction. Reconstructed images had an image matrix size of 144×144, a voxel size of 4×4 mm and a slice thickness of 5 mm.

All patients underwent the standard [^18^F]FCH image acquisition protocol at our institution: following the LD-CT, ‘early’ PET image acquisition started 2 min after intravenous injection of 4 MBq/kg [^18^F]FCH, using a 35 cm scan trajectory over the pelvic region (2 min/bed position); patients were asked to void 20 min post injection (p.i.), and at 30 min p.i. a ‘late’ whole body PET sequence was started, from mid-thigh to the skull vertex, again using 2 min acquisitions/bed position. Patient preparation was similar to that required for FDG PET [28].

### PET-CT Data Analysis

PET-CT images were evaluated by an experienced nuclear medicine specialist who first identified all lymph nodes with enhanced FCH uptake versus their direct background (on early and/or late scan time points) within the field of view of the early scan trajectory. Lymph node diameters were measured using the CT component of the PET-CT scanner, and standard CT and MRI, where available.

PET and LD-CT images were converted to ECAT 7 format and regions of interest (ROIs) were semi-automatically drawn around every pelvic LN that met the inclusion criteria mentioned above, using in-house developed software, as previously described [29], [30]. For lesion delineation, we used the adaptive threshold of 50% of maximum voxel value within tumor, the 3D volume of interest A50 (VOIA50). This method is similar to the fixed threshold method, except that it adapts the threshold relative to the local average background, thereby correcting for the contrast between tumour and local background. For example, the A50 contour value corresponds to a value at 50% of the sum of the maximum voxel value and the local background value. The latter value is derived from ‘background’ voxels that are identified as those voxels located on a single voxel thick shell at 2.5 cm from the edge of a 70% of maximum pixel value isocontour, excluding all voxels with an SUV larger than 2.5.

SUV’s were normalized for body weight. For data-analysis, we used early (2 min p.i.) and late (30 min p.i.) SUV_max_ and SUV_mean_, as well as their absolute [SUV late - SUV early] and relative differences [(SUV late - SUV early)/SUV early].

Furthermore, based on literature [8], we considered two possible time-trends of [^18^F]FCH uptake by comparing the SUV early and the SUV late: type A pattern (decreasing over time) if the SUV early > the SUV late and type B pattern (stable/increasing over time) if the SUV late was equal or exceeded the SUV early. Equality of SUV’s was decided using the 2^nd^ decimal. In the present context, accuracy measures relate to the ability of time-trends (2 versus 30 min p.i.) of tracer uptake to discriminate malignant and benign lymph nodes with enhanced [18F]FCH uptake.

### Statistical Analysis

The two sample Mann-Whitney tests were performed to determine a shift in the median values for benign and malignant tumors. Linear mixed-effects models were constructed to determine the relation between SUV_max_ and SUV_mean_. Included in this model were tumor status (benign versus malignant) and post injection scan time (2 min versus 30 min) as fixed effects, as well as random slopes per patient and per lesion (nested within patient). The residuals were assumed to be exponential related to SUV_mean_, and pairwise interactions between SUV_mean_ and both tumor status and scan time were also tested. An identical linear regression model ignoring repeated measures and heteroscedasticity was used to provide an R^2^. Receiver Operating Characteristic (ROC) curves were constructed to determine the thresholds maximizing specificity and sensitivity. The binomial distribution is used to determine 95% confidence intervals of the sensitivity and specificity estimates for the optimal thresholds. To ensure that these results are not influenced by within-patient correlations in uptake, a Monty Carlo process is performed where ROC curves are produced for 500 datasets randomly generated so that each patient has only one lesion. The range of the sensitivities and specificities of the optimal thresholds from these ROC curves were visually compared with the sensitivities and specificities calculated using all observations.

## Results

We identified 25 eligible patients who had 54 lymph nodes with enhanced [18F]FCH uptake that met our inclusion criteria. In 13/25 (52%) patients the PET-CT had been performed because of PSA relapse (I_1_), and in 9/25 (36%) for staging at presentation (I_2_); the remaining three patients had been referred for restaging in the context of presumed oligometastases (I_3_). The mean interval between the primary therapy and the time of referral to [^18^F]FCH PET-CT was 23 months (range: 3–48 months). In 17 patients (pelvic group; median age: 63 years; range: 50–80 years) we found 34 enlarged pelvic nodes (classified as malignant, see methods), and there were 7 patients (inguinal group; median age: 63 years; range: 57–68 years) with 15 [^18^F]FCH positive inguinal LN, classified as benign. One patient had enhanced [^18^F]FCH uptake in both two inguinal and three enlarged pelvic nodes. In either group the median number of eligible lymph nodes per patient was 2 (range: 1–6). Patient characteristics are presented in Table 1.

All LN showed enhanced FCH uptake at early (2 min p.i.) as well as late (30 min p.i.) time points (Table 2). The long axes of the inguinal and pelvic lymph nodes were similar (11±2, and 12±3 mm, respectively); short axis diameters were slightly smaller in the former group (8±1 vs. 10±2 mm, respectively; *P*<0.001).

We found highly significant associations between the LN status (inguinal/benign vs. enlarged pelvic/malignant) and the SUV_max_ and SUV_meanA50_ 30 min p.i., and their absolute and relative differences (*P*<0.0001). The correlation between the mean and max SUV metrics was near-perfect (Fig. 1; the linear regression model resulted in an almost identical relation and an adjusted R^2^ of 0.99). For further analyses we focused on the SUV_max_. The number of LN with a [^18^F]FCH uptake pattern type A (SUV_early_ > SUV_late_) or B (SUV_early_ ≤ SUV_late_), in the benign and malignant group for the above mentioned thresholds are included in Table 3. Based on the SUV_max_ relative difference, all but one LN in the benign group showed a type A, decreasing uptake over time, while in the malignant group 95% (35/37) of the nodes presented a type B pattern uptake. From the 54 LN, only 3 nodes were found to have a stable uptake over time: one from the inguinal and two from the pelvic group, respectively.

**Figure 2:**
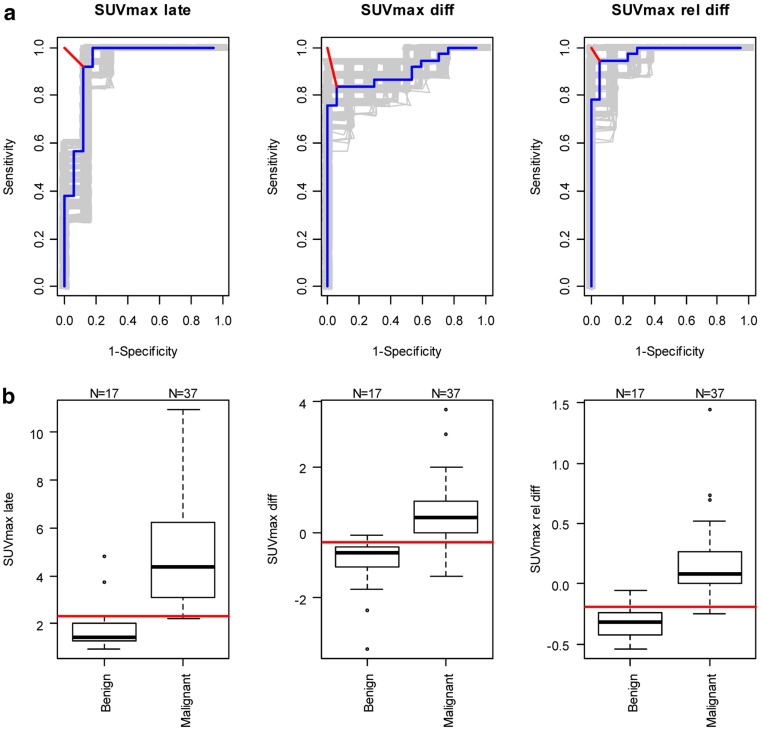
ROC analyses and Treshold boxplots. a ROC analyses of SUV_max_ 30 min p.i. assessment (late), difference (SUV_late_ – SUV_early_) and relative difference [(SUV_late_ – SUV_early_)/SUV_early_]. The grey lines represent the 500 ROC curves for randomly generated datasets in which only one lesion is included for each patient; the blue lines are the ROC curves for all 54 lesions; the red lines indicate the shortest distance to the top left hand corner; **b** Threshold boxplots. Thresholds associated with the shortest distance to the top left hand corner in the ROC curves are indicated in the boxplots by the horizontal red lines.

ROC analyses of uptake trends over time and of SUV’s at either time-point (Fig. 2) showed that the SUV_max_ relative difference was the best predictor of the lymph node status, followed by the SUV_max_ late and the SUV_max_ absolute difference [see also Table 3 for the areas under the ROC curve (AUC)]. The threshold of SUV_max_ relative difference that maximized both sensitivity and specificity was −0.19, for a sensitivity of 95% (95%CI 82–99) and a specificity of 94% (95%CI 71–100), versus a SUV_max_ late threshold of 2.3, for a sensitivity of 92% (95%CI 78–98) and a specificity of 88% (95%CI 64–99).

In the 17 patients with 34 enlarged [^18^F]FCH positive pelvic nodes, histopathological confirmation was obtained in 4 (11 LN); all had type B time trends at [^18^F]FCH PET-CT. During follow-up, 3 patients (6 LN) had radiological nodal progression; from these five LN had a type B pattern and one LN proved to be false negative: type A trend and radiological progression. In 3 other patients, with only pathologically enlarged pelvic lymph nodes (6 LN) to explain an elevated PSA and with a type B pattern, PSA normalized upon therapy, accompanied by shrinkage of these nodes. PSA decrease and disappearance of a solitary (type B) pelvic node was observed in another patient who was treated with pelvic radiotherapy that focused on a suspected recurrence in prostate and this lymph node.

**Figure 3:**
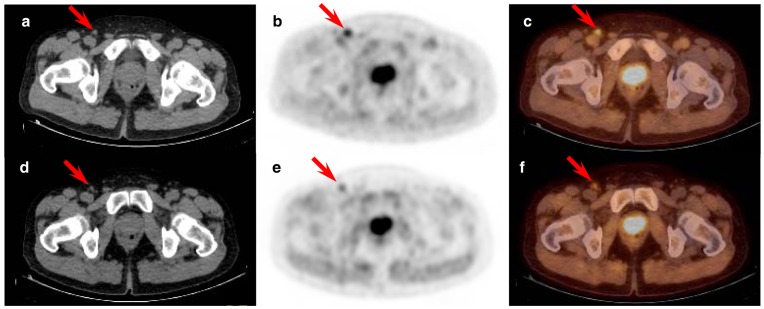
Example of decreasing [18F]FCH uptake over time. Example of decreasing [18F]FCH uptake over time in a right inguinal lymph node (red arrow; SUV_max_ early: 4.41; SUV_max_ late: 2.03) of a patient with newly diagnosed prostate cancer. This lymph node was classified as benign. Transversal sections of the Low-dose CT, PET and fused PET-CT images: **a**, **b** and **c** – early phase; **d**, **e** and **f** – late phase. [18F]FCH uptake in the prostate is also visible.

**Figure 4:**
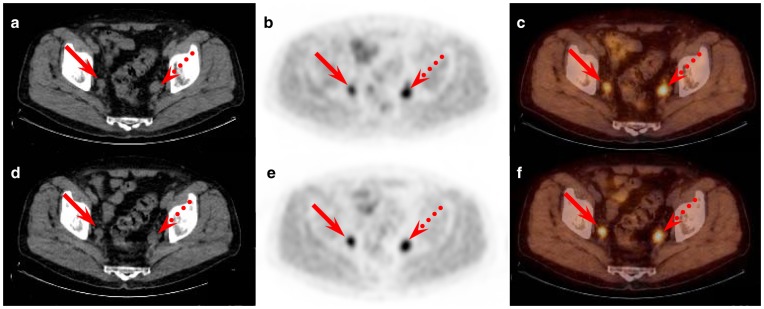
Example of increasing [18F]FCH uptake over time. Example of increasing [18F]FCH uptake over time in a right para-iliac lymph node (red solid arrow; SUV_max_ early: 3.02; SUV_max_ late: 4.35) and a left para-iliac node (red dotted arrow; SUV_max_ early: 3.47; SUV_max_ late: 4.79) of a patient with biological relapse after initially treated prostate cancer. These lymph nodes were classified as malignant. Transversal sections of the Low-dose CT, PET and fused PET-CT images: **a**, **b** and **c** – early phase; **d**, **e** and **f** – late phase.

Six other patients (with 9 type B and 1 type A pelvic lymph nodes) were treated with systemic therapy, because of local recurrence and/or skeletal metastases. Finally, the patient with both 2 inguinal and 3 pelvic LN (type A in the inguinal and type B in the pelvic nodes) was treated with RT (prostate) and ADT because of confirmed bone oligometastases at presentation. In these 7 patients, PSA decreased over time and radiological regression of all 13 enlarged pelvic nodes together with stable dimensions of the inguinal nodes were recorded, as well. However, this did not unequivocally confirm the LN status due to the use of systemic therapy for coexistent bone metastases.

Of the inguinal nodes (14 type A and 1 type B with stable uptake over time) none showed signs of malignancy during follow-up; of these, in one patient a lymph node biopsy was tumor negative. One patient (2 LN) opted for watchful waiting policy (otherwise negative PET), and his PSA was stable at 0.4 ng/ml for the follow-up of 12 months. In another patient (type A LN) a local recurrence was treated with HIFU with good clinical response (PSA) and no signs of progression.

## Discussion

In our referral-based spectrum of patients with enhanced [^18^F]FCH uptake in pelvic and inguinal lymph nodes, decreasing [^18^F]FCH uptake over time seems to be a reliable tool to differentiate benign and malignant nodes. Together with similar findings by others to classify radioactive choline positive lesions suspected to represent hematogeneous metastases, our results are relevant for clinical decision making and simplification of diagnostic procedures, eg. in patients with elevated PSA and positive [^18^F]FCH PET findings. Moreover, the results underline the relevance of a sequential PET imaging protocol after a single injection of [^18^F]FCH to account for the time-trend of tracer uptake.

We classified disease-status as *malignant* for enlarged pelvic nodes, and as *benign* for inguinal nodes of any size. Our criterion of benignity was based on the typical prostate drainage pattern which does not include inguinal nodes, as described by Inoue et al. [20]. They identified by using fluorescence navigation 3 lymphatic drainage pathways, comprising the obturator, the external and internal iliac nodes. Similar drainage patterns were found by Tokuda et al. [21] in 125 patients with LN metastases. Weckermann et al. [22] performed both sentinel lymph node dissection and radical prostatectomy in 1055 patients with PCa. Despite a high percentage (>50%) of positive nodes identified outside the standard lymphadenectomy borders, none of them were found in the inguinal region. In our study we also never encountered occurrences of malignancy in inguinal nodes (histological analysis, clinical radiological follow-up).

We considered pelvic nodes with a short axis diameter equal or exceeding 8 mm as being malignant. This threshold was chosen based on the study of Jager et al. [23] who reported a 98% specificity for MRI using this dimension. In their meta-analysis, Hövels et al. [31] found that false positivity of CT/MRI (similar performance for either technique) at thresholds of 8–10 mm is only 7%. In an attempt to reduce the remaining uncertainty, a standard of reference method was used. This approach, as extensively described in the “Materials and Methods” section, consisted of the combination of histopathological examination (whenever available) and the results obtained by clinical or radiological follow-up. This is a commonly used procedure [32]–[37] to account for the limitations of retrospective studies. In difficult cases, biopsy of the proper radioactive choline avid lymph nodes was improved and verified by using a dedicated gamma-probe [38]. Confirmation seemed feasible in 65% of these pelvic LN (24**/**37). In 7 patients treated with ADT and/or chemotherapy for coexisting bone metastases, decreases of nodal diameter could not be interpreted since such changes are not necessarily compatible with a ‘malignant tissue’ response to treatment.

Note that in our present context ‘sensitivity’ and ‘specificity’ should not be confused with ‘the accuracy of [^18^F]FCH PET-CT to diagnose metastatic lymph nodes in prostate cancer’. The results pertain to the ability of tracer uptake time-trends to classify lymph nodes with enhanced [^18^F]FCH uptake.

The relevance of uptake time-trends to characterize [^18^F]FCH foci has been demonstrated in malignant bone metastases, in recurrent PCa, and in malignant zones of the prostate in preoperative setting [8], [15], [17]. Our findings corroborate and extend those of Beheshti et al. [8] who reported on 18 malignant lymph nodes showing stable or increasing uptake over time. The imaging protocol consisted of a dynamic PET/CT scan of the pelvic region for 8 min, starting 1 min p.i., followed by whole body (WB) images 10 min after [^18^F]FCH injection and optional supplementary delayed WB acquisitions, 90–120 min p.i., when abnormalities were detected. However, since that study comprised only 4 [^18^F]FCH positive reactive lymph nodes (with decreasing uptake over time) they urged for validation of these patterns in a larger study.

In our study, all but one inguinal nodes showed decreasing [^18^F]FCH uptake over time (Fig. 3), versus 95% (35/37) of the pelvic category demonstrating stable or increasing uptake (Fig. 4). Kwee et al. [15] suggested as a possible explanation for the tracer decrease over time in benign zones the dephosphorylation of [^18^F]FCH by prostatic acid phosphatase while a trapping mechanism of the tracer in the malignant cells was responsible for the increased uptake in PCa. This can only be validated with full kinetic modeling.

Our results are at variance with those of Cimitan et al. [15] who reported no significant late/early [^18^F]FCH uptake ratios in proven local recurrent prostatic disease or abdominopelvic LN, when performing a dual phase [^18^F]FCH PET-CT in 43 patients with PSA relapse. However, their dual-phase PET/CT protocol included WB scans with variable early and late acquisitions, 5 to 15 min p.i. and 65 to 200 min p.i., respectively. The discrepancy may be explained by the rapid clearance of the [^18^F]FCH after administration [14], [16], which implies that timing of the early acquisitions is crucial and should be done using a strict imaging protocol.

The prevalence of patients with inguinal lymph nodes showing enhanced [^18^F]FCH uptake was 12% (8/66 patients). Our finding that reactive nodes remained detectable over time (i.e., for 30 min. after injection) seems being inconsistent with the observation of Price et al. [16] who found persistent uptake at the late, 45 min p.i., images in only 1 of 4 patients with initially enhanced inguinal node uptake. We content that this variance results from improved signal to noise ratios with the current TOF scanner generations.

Our data suggest that the type B pattern is a strong indication for malignancy, the PPV being 97% (35/36; see Table 3, for the best LN status predictor: SUVmax Relative Difference). The fact that the PPV is not 100% implies in clinical practice that, e.g. in case of multiple potentially malignant [^18^F]FCH positive LN, the ones with type B patterns should be the primary biopsy candidates.

Our results also suggest that a single point measurement in the context of a whole body scan, starting in caudocranial direction, 30 min after injection, is a reasonable alternative for relative change to differentiate reactive from malignant LN in patients with PCa. Obviously, omitting the early scan would simplify the scan procedure. However, compared to measuring relative changes, the use of absolute SUV’s is more demanding at the level of standardization. Evidently, the present results pertain to lymph nodes with enhanced [^18^F]FCH uptake and ≥8 mm short axis. Ascertaining the single time-point approach requires validation versus histopathology.

### Conclusion

Time-trends of enhanced [^18^F]FCH uptake in lymph nodes of prostate cancer patients seem to help discriminate benign from malignant localizations. Single time-point SUV measurements, 30 min p.i., may be a reasonable alternative for predicting the nodal status, but this remains to be validated in non-enlarged pelvic lymph nodes.
